# Do airway inflammation and airway responsiveness markers at the start of apprenticeship predict their evolution during initial training? A longitudinal study among apprentice bakers, pastry makers and hairdressers

**DOI:** 10.1186/s12890-018-0674-9

**Published:** 2018-07-11

**Authors:** Valérie Demange, Denis Zmirou-Navier, Abraham Bohadana, Pascal Wild

**Affiliations:** 1 Institut National de Recherche et de Sécurité (INRS), 1 rue du Morvan, 54519 Vandoeuvre-les-Nancy, France; 2 School of Public Health (EHESP), 15 avenue du Professeur Léon-Bernard, 35043 Rennes Cedex, France; 3 Pulmonary Institute, Shaare Zedek Medical Center, 12 Baiyt Street, 91031 Jerusalem, Israel

**Keywords:** Occupational asthma, Airway inflammation, Fractional exhaled nitric oxide, Bronchial challenge test, Airway resistance, Apprentices, Bakers, Hairdressers

## Abstract

**Background:**

The natural history of airway inflammation and symptoms in occupations at risk of asthma is still not fully understood. We aimed to study the evolution during apprenticeship of inflammation markers, bronchial hyperresponsiveness (BHR) and symptoms in at-risk subgroups as defined from measurements of markers made shortly after the start of training.

**Methods:**

Respiratory symptoms, FEV_1_ and airway resistance post-bronchial challenge (MBC) test results, fractional exhaled nitric oxide (FeNO) measurements, and eosinophils in nasal lavage fluid were investigated in apprentice bakers, pastry-makers and hairdressers. Four visits were conducted: at the start of the training and every six months thereafter. Four baseline risk groups were defined, based on, (i) a high level of FeNO (NO), (ii) eosinophils > 1% (Eosino), (iii) a ≥ 15% decrease in FEV_1_ during the MBC test (HR), and (iv) a ≥ 50% increase in the resistance (Resist). The statistical analysis relied on mixed models.

**Results:**

At baseline, the inflammation markers were related to the MBC markers. There was no evidence to suggest that the baseline risk groups predict a differential evolution of the airway inflammation and bronchial responsiveness markers, or the asthma-like symptoms considered. The baseline risk groups defined from MBC test predicted the levels of MBC markers. Similarly, the baseline risk groups based on eosinophilic inflammation predicted the levels of markers for eosinophilia. These results were similar in the three training tracks, with the exception of the FeNO levels which were not different according to the Eosino risk group. Twelve possible new asthma cases were identified, only the HR risk group predicted their occurrence.

**Conclusions:**

Among this young population, at-risk groups based on initial high levels of inflammation markers did not experience any worsening during the follow-up. However, initial BHR predicted consistently high levels of all markers considered and occurrence of possible asthma.

**Electronic supplementary material:**

The online version of this article (10.1186/s12890-018-0674-9) contains supplementary material, which is available to authorized users.

## Background

Occupational asthma (OA), defined as “asthma caused by exposure to sensitizing agents and/or irritants in the workplace” [1], is one of the most common occupational diseases of the lung in industrialised countries [2]. In France, incidences of OA in bakers and pastry-makers (683 cases/million subjects) and in hairdressers (308/million) are respectively the highest and the third highest of all occupations [3]. More data are available about work-related asthma, a term that refers to “asthma both exacerbated or induced by inhalation exposures in the workplace” [1]. Although there was an overall decrease of work-related asthma in France over the period 2001–2009, different temporal trends in work-related asthma associated with flour can be observed depending on the sector considered; for example near significant decreases in the incidence of work-related asthma were observed in industrial settings and in sales sectors, but not in the catering industry [2]. No statistically significant temporal trend for this period was observed in work-related asthma linked to hairdressing products [2].

From a pathological standpoint, OA, like non-occupational asthma, is characterized by acute and chronic inflammation of the airways [4], with an increased presence of eosinophils and/or neutrophils in the lower airways compared to the levels observed in non-asthmatics [5]. Some studies of OA patients have observed differential neutrophil and eosinophil cell counts in induced sputum as a function of the molecular weight of the causal agent [6–8]. However, there are some discrepancies in these patterns: more eosinophils in cases of High Molecular Weight (HMW) agents [7, 8], more eosinophils in cases of Low Molecular Weight (LMW) agents [6], as well as non-differential cell counts regardless of the molecular weight [9]. The effects of exposure may start before employment, i.e. during training, as indicated in two studies of apprentices on different training programmes [1, 10]. An apprenticeship might even represent a period during which an individual is more vulnerable to the noxious effects of HMW agents than they are during employment [11].

In a previous paper, we reported data from a cohort of non-asthmatic apprentices recruited at the beginning of training in occupations associated with a risk of asthma [12]. With this cohort, we aimed to investigate the early development of OA, deliberately excluding any subject with a suspicion of asthma. Work-exacerbation of previously existing asthma is out scope for this cohort. This follow-up included monitoring of airway inflammation and asthma-like symptoms in apprentice bakers, pastry-makers and hairdressers. We found, first, that increase in FeNO between visits predicted the incidence of BHR [13]. Second, we were able to identify, at the start of apprenticeship, a subgroup of subjects with eosinophilic inflammation (the highest values of FeNO and eosinophils in nasal lavage fluid) [14] that could potentially lead to asthma.

The primary objective of the present paper was to assess whether the evolution of inflammation markers, bronchial hyperresponsiveness and respiratory symptoms during the apprenticeship is indeed different in subgroups potentially at an early stage of asthma, identified using markers at the baseline. A secondary objective was to investigate whether the evolution of these markers, in particular the eosinophilic markers, differed according to the occupation; bakers and pastry makers, unlike hairdressers, being exposed to HMW agents.

## Methods

All apprentice bakers, pastry-makers and hairdressers at the start of training programmes in six vocational schools in Lorraine, North-Eastern France, were invited to take part in the research [12]. 1839 apprentices from 6 apprenticeship schools were approached to take part in the study and 1399 refused or did not meet the inclusion criteria [12]. Volunteers were included by physicians if they had neither a history of previous occupational exposure to substances known to induce OA nor any suspicion or history of asthma. To avoid any risk of including apprentices with asthma, we further excluded post-hoc 2 subjects who reported both at least an asthma-like symptom and showed BHR.

Four visits were conducted in total: a visit at the start of the apprenticeship and then one every 6 months until completion of the training. The study was approved by the local ethical committee (namely “Comité Consultatif de Protection des Personnes participant à une Recherche Biomédicale” from Lorraine, n°02.09.02) and written consent was obtained from either the apprentices or their parents, depending on the age of the apprentice. In total, 441 apprentices participated, representing 24% of the eligible apprentices. As this paper focuses on the evolution of markers during apprenticeships, we retained all apprentices who completed the first visit and at least one other visit (318 apprentices).

### Questionnaire

Symptoms were assessed using a standard questionnaire [15]. Rhinoconjunctivitis-like symptoms were defined as itchy, runny, stuffy nose or sneezing, and/or red, burning or weeping eyes, except during a respiratory infection, occurring irregularly either almost every day or from time to time. Asthma-like symptoms were defined as wheezing, chest tightness, shortness of breath or coughing, except during a respiratory infection or during exercise. Atopy is a personal or familial tendency to produce IgE antibodies in response to ordinary exposure to allergens, and to develop typical symptoms such as asthma, rhinoconjunctivitis, or eczema [16]. We defined “atopic disease” in cases of hay fever and/or eczema and/or asthma in childhood and “atopic disposition” in cases of asthma or allergy in siblings and/or parents.

### Fractional exhaled nitric oxide (FeNO)

FeNO was measured according to ATS/ERS recommendations [17] using a chemiluminescence analyzer (NIOX® 2.0 system; Aerocrine AB, Solna, Sweden). FeNO was expressed as observed parts per billion (ppb).

### Pulmonary function tests

Pulmonary function tests were undertaken after measurement of FeNO, using a Random-noise Oscillatory Spirometer, combining respiratory impedance by Forced Oscillation Technique (FOT) and spirometric expiration measurements (SensorMedics Corporation, Datalink, Montpellier, France). Total impedance of the respiratory system by FOT was systematically measured prior to spirometry to avoid undesirable effects of forced expiratory maneuvers. A mean oscillatory resistance of 4 to 16 Hz (Rrs4–16) was used as the outcome variable [12]. Spirometry was performed from the sitting position. At least three baseline forced expiratory manoeuvres meeting recommended criteria [18] were conducted, the subject expiring forcefully after a maximum inspiratory maneuver. The highest forced vital capacity (FVC) and forced expiratory volume in one second (FEV_1_) values were recorded for analysis. FEV_1_ values were expressed in percent of predicted values according to sex, age and height [18].

Non-specific airway responsiveness was assessed using the methacholine bronchial challenge (MBC) test based on the procedure described in [19]. Three cumulative doses of methacholine (100, 600 and 1600 μg, respectively) were administered in succession [12]. The test was finished either after administering the last dose of methacholine or when the FEV_1_ decreased by 20% or more below the baseline value. In this case, the subjects were considered MBC+ 20%. The decrease in FEV_1_ during the MBC test was defined as the difference between the FEV_1_ at the highest methacholine dose and the baseline FEV_1_, divided by the baseline FEV_1_. The increase in resistance post-MBC test was defined as the difference between the maximum Rrs4–16 after methacholine inhalation increase and the baseline Rrs4–16, divided by the baseline Rrs4–16.

### Eosinophil count in nasal lavage fluid

Nasal lavage was performed using the Hilding’s method [20]. Slides containing > 30% squamous cells were rejected. Cells were counted and the corresponding eosinophil percentages (EP) were determined.

### Skin prick tests

Skin Prick Tests (SPT) were performed using dust mites, animal danders, pollens and moulds (Stallergenes Laboratories, Fresnes, France; ALK-Allerbio Laboratory, Varennes en Argonne, France). A positive SPT was defined as a wheal diameter 3 mm or more greater than obtained with the negative controls after 20 min [12]. Personal atopy was defined as the presence of a positive response to at least one common allergen [21]. The degree of sensitization, defined as the number of positive responses to the common allergens in three classes (0, 1 to 2, ≥3), was also used [22].

### Definition of possible asthma cases

We defined possible asthma cases if the subjects reported asthma-like symptoms and showed 15% decrease in FEV_1_ during the MBC test. We did not consider only one of these two criteria as sufficient to define a possible asthma. OA was defined as the combination of possible asthma and occupational sensitization.

### Baseline risk groups

In this paper, we considered four at-risk groups: (i) the NO group, defined by a baseline FeNO level higher than 27 ppb; (ii) the Eosinophil Count (Eosino) group, defined by an EP greater than 1% in the nasal lavage; (iii) the Airway Responsiveness FEV_1_ (HR) group, defined by a 15% or more decrease in FEV_1_ during the MBC test; and (iv) the Airway Responsiveness Resistance (Resist) group, defined by a 50% increase in resistance between 4 and 16 Hz.

We chose a baseline FeNO level of higher than 27 ppb to define the NO group and a threshold of 15% instead of the usual 20% for the decrease in FEV_1_ for the HR group so that the NO+ and HR+ subgroups would be of similar size to the other at-risk subgroups. The threshold of 27 ppb is close to the threshold of 25 ppb, which is clinically used to indicate in asthmatic patients that the eosinophilic inflammation is unlikely [23]. The level of 15% of the decrease in FEV_1_ enables us to be more sensitive in the detection of airway hyperresponsiveness.

### Statistical analyses

Statistical analyses were conducted using the Stata 15.1 package (Stata, College Station, TX, USA). The evolution of the different markers (asthma-like and rhinoconjunctivitis-like symptoms, possible asthma, airway responsiveness and resistance, eosinophil count and FeNO) was modelled using a mixed model with the subject as a random effect, in order to account for within-subject correlations. When modelling a continuous outcome (e.g. the FeNO level), a linear model was applied, whereas when considering a binary outcome (e.g. a nasal eosinophil count greater than 1%), a logistic model was applied. The risk groups at the baseline visit were included as independent variables in addition to the interaction terms between each group and the visit, to enable assessment of a possible differential evolution over time as a function of the baseline risk groups. All analyses were adjusted for relevant outcome-specific potential confounders without any selection on statistical significance. When analysing evolution of rhinoconjunctivitis, asthma-like symptoms, possible asthma, eosinophil count ≥1%, the confounders were sex, degree of sensitization, tobacco usage status, training track, and visit. When analysing the decrease of FEV_1_ during MBC test, increase of resistance during MBC test, FeNO level, the confounders were sex, height, degree of sensitization, tobacco usage status, training track, and visit. When the outcome considered was used in the definition of the risk group, the baseline visit was excluded. For example, when analyzing the evolution of FeNO as a function of FeNO, we considered only visits 2, 3 and 4, whereas when analyzing FeNO as a function of Resistance or BHR, we considered all four visits. The results are given in terms of the *p*-value for the interaction between the baseline risk group and the visit. When this interaction proved not statistically significant, the model was refitted without the interaction and we thus report the differences between the subjects at risk and the subjects not at risk as defined by the baseline risk group.

## Results

The ages of the 318 participants ranged from 15.3 to 24.8 years, with a median age of 16.6 years (data not shown). Table 1 presents the associations between the risk groups and the personal characteristics at baseline. Personal atopy was found to be significantly more frequent among the subjects of the at-risk groups than it was among the other subjects. Surprisingly, there were more subjects with an atopic disease in the Eosino- group than in the Eosino + group (20% versus 7%, *p* = 0.04). No other personal characteristic showed a statistically significant association with any of the risk groups. Eighty-seven percent of the apprentices had a FEV_1_ in percent predicted to be over 80%. In the Additional file 1, the same characteristics at baseline are presented according to the training track.

**Table 1:** Baseline association between risk groups and the personal characteristics of the 318 subjects (median [interquartile range], % (n))

	NO group		Eosinophil count group		Airway responsiveness FEV_1_ group		Airway responsiveness resistance group	
	+^a^ *n* = 46	-^a^ *n* = 272	+^b^ *n* = 44	-^b^ *n* = 274	+^c^ *n* = 44	-^c^ *n* = 274	+^d^ *n* = 27	-^d^ *n* = 291
Sex: male 55.3% (176)	58.7% (27)	54.8% (149)	63.6% (28)	54.0% (148)	56.8% (25)	55.1% (151)	55.6% (15)	55.3% (161)
	*p* = 0.62		*p* = 0.23		*p* = 0.83		*p* = 0.98	
Atopic disposition 37.1% (118)	41.3% (19)	36.4% (99)	47.7% (21)	35.4% (97)	47.7% (21)	35.4% (97)	33.3% (9)	37.5% (109)
	*p* = 0.52		*p* = 0.12		p = 0.12		*p* = 0.67	
Atopic disease 18.2% (58)	15.2% (7)	18.8% (51)	**6.8% (3)**	**20.1% (55)**	15.9% (7)	18.6% (51)	22.2% (6)	17.9% (52)
	*p* = 0.57		**p = 0.04**		*p* = 0.83		*p* = 0.60	
Personal atopy based on SPT								
≥ 1 positive response 32.1% (102)	**67.4% (31)**	**26.1% (71)**	**52.3% (23)**	**28.8% (79)**	**54.6% (24)**	**28.5% (78)**	**51.9% (14)**	**30.2% (88)**
	***p*** **< 0.0005**		***p*** **= 0.002**		***p*** **= 0.001**		***p*** **= 0.020**	
Degree of sensitization								
0 positive response	**32.6% (15)**	**68.5% (185)**	**39.5% (17)**	**67.0% (183)**	**45.5% (20)**	**66.2% (180)**	44.4% (12)	65.1% (188)
1 to 2 positive responses	**21.7% (10)**	**19.6% (53)**	**25.6% (11)**	**19.1% (52)**	**25.0% (11)**	**19.1% (52)**	29.6% (8)	19.0% (55)
≥ 3 positive responses	**45.7% (21)**	**11.9% (32)**	**34.9% (15)**	**13.9% (38)**	**29.6% (13)**	**14.7% (40)**	25.9% (7)	15.9% (46)
	***p*** ** < 0.0005**		***p*** ** = 0.001**		***p*** **= 0.017**		*p* = 0.10	
Training track								
Bakery- Pastry making 60.1% (191)	69.6% (32)	58.5% (159)	65.9% (29)	59.1% (162)	54.6% (24)	61.0% (167)	74.1% (20)	58.8% (171)
Hairdressing 39.9% (127)	30.4% (14)	41.5% (113)	34.1% (15)	40.9% (112)	45.5% (20)	39.1% (107)	25.9% (7)	41.2% (120)
	*p* = 0.16		*p* = 0.39		*p* = 0.42		p = 0.12	
Tobacco usage status								
Non smoker 49.7% (158)	63.0% (29)	47.4% (129)	54.6% (24)	48.9% (134)	47.7% (21)	50.0% (137)	33.3% (9)	51.2% (149)
Current smoker 46.2% (147)	32.6% (15)	48.5% (132)	43.2% (19)	46.7% (128)	50.0% (22)	45.6% (125)	63.0% (17)	44.7% (130)
Past smoker 4.1% (13)	4.4% (2)	4.0% (11)	2.3% (1)	4.4% (12)	2.3% (1)	4.4% (12)	3.7% (1)	4.1% (12)
	*p* = 0.13		*p* = 0.69		*p* = 0.74		*p* = 0.18	
Baseline FEV_1_								
> 80.0% 87.1% (277)	91.3% (42)	86.4% (235)	81.8% (36)	88.0% (241)	**79.6% (35)**	**88.3% (242)**	92.6% (25)	86.6% (252)
> 70.0 and < 80.0% 12.3% (39)	8.7% (4)	12.9% (35)	15.9% (7)	11.7% (32)	**15.9% (7)**	**11.7% (32)**	7.4% (2)	12.7% (37)
< 70.0% 0.6% (2)	0	0.7% (2)	2.3% (1)	0.4% (1)	**4.6% (2)**	**0**	0	0.7% (2)
	*p* = 0.73		*p* = 0.16		***p*** **= 0.012**		*p* = 0.63	

^a^NO Group +: subjects with a baseline FeNO level > 27 ppb; −: subjects with a baseline FeNO level < 27 ppb

^b^Eosinophil Count Group +: subjects with a baseline percentage of eosinophils > 1% in the nasal lavage; −: subjects with no eosinophils at baseline

^c^Airway Responsiveness FEV_1_ Group +: subjects with a baseline decrease in FEV_1_ of 15% or more during the MBC test; −: subjects with a baseline FEV_1_ decrease < 15%

^d^Airway Responsiveness Resistance Group +: subjects with a baseline increase in resistance of 50% or more between 4 and 16 Hz; −: subjects with a baseline increase in resistance of < 50%

*p*: *p*-value of the test comparing the presence or level of a marker in the +risk group and -risk group

As expected, all eosinophilic markers at baseline were significantly different according to the two eosinophilic baseline risk groups (NO and Eosino groups) (Table 2). Similarly, all airway responsiveness markers at baseline were significantly different according the two baseline risks groups based on MBC test (HR and Resist groups). Somewhat less expected are the significant differences of the FEV_1_ decline in the MBC test between the two categories among the NO group and in FeNO levels between the baseline risk groups based on the MBC test (HR and Resist).

**Table 2:** Baseline associations between risk groups and airway inflammation and responsiveness markers among the 318 subjects (median [interquartile range], % (n))

	NO group		Eosinophil count group		Airway responsiveness FEV_1_ group		Airway responsiveness resistance group	
	+^a^ *n* = 44	-^a^ *n* = 274	+^b^ *n* = 44	-^b^ *n* = 274	+^c^ *n* = 44	-^c^ *n* = 274	+^d^ *n* = 27	-^d^ *n* = 291
Rhinoconjunctivitis-like symptoms	6.5% (3)	9.2% (25)	9.1% (4)	8.8% (24)	4.6% (2)	9.5% (26)	11.1% (3)	8.6% (25)
	*p* = 0.78		*p* = 1.00		*p* = 0.40		*p* = 0.72	
Asthma-like symptoms	6.5% (3)	1.8% (5)	4.6% (2)	2.2% (6)	0.0% (0)	2.9% (8)	3.7% (1)	2.4% (7)
	*p* = 0.09		*p* = 0.31		*p* = 0.61		*p* = 0.51	
MBC test								
(Pre – post-test)/pretest FEV_1_[%]	**−9.9 [− 14.8;-5.4]**	**−5.8 [− 10.0;-3.1]**	−7.5 [− 12.2;-3.6]	−6.2 [− 11.2;-3.6]	*−18.9 [− 26.0;-17.2]*	*−5.5 [− 8.7;-2.9]*	**−9.8 [− 16.7;-7.9]**	**−5.8 [− 11.0;-3.4]**
	***p*** **= 0.0009**		*p* = 0.59		^e^		***p*** **= 0.0015**	
(Post– pretest)/pretest Rrs4–16 [%]	17.7 [10.7;34.0]	15.8 [6.5;29.0]	14.4 [5.3;28.3]	16.1 [6.8;29.7]	**24.9 [12.7;37.2]**	**15.0 [6.4;26.4]**	*63.6 [53.0;77.2]*	*14.6 [6.4;24.1]*
	*p* = 0.16		*p* = 0.63		***p*** **= 0.0017**		^e^	
Eosinophil count								
Eosinophil count < 1 86.2% (274)	**69.6% (32)**	**89.0% (242)**	*–*	*100.0% (274)*	81.8% (36)	86.9% (238)	88.9% (24)	86.0% (250)
	***p*** ** < 0.0005**		^e^		*p* = 0.35		*p* = 1.0	
Eosinophil count [%] if ≥1%	**32.5 [15.0;60.0]**	**9.5 [3.6;20.0]**	*13.9 [5.2;30.9]*	*–*	37.0 [15.1;65.7]	11.9 [4.9;23.3]	60.0 [16.7;80.0]	12.0 [5.0;26.6]
	***p*** **= 0.006**		^e^		*p* = 0.063		*p* = 0.07	
FeNO [ppb]	*45.4 [34.0;64.1]*	*11.8 [8.7;16.0]*	**17.0 [10.2;45.5]**	**12.3 [9.0;17.4]**	**17.2 [10.4;30.7]**	**12.3 [8.9;17.6]**	**17.2 [11.0;33.2]**	**12.5 [9.0;17.9]**
	^e^		***p*** **= 0.0029**		***p*** **= 0.0042**		***p*** ** = 0.02**	

^a^NO Group +: subjects with a baseline FeNO level > 27 ppb; −: subjects with a baseline FeNO level < 27 ppb

^b^Eosinophil Count Group +: subjects with a baseline percentage of eosinophils > 1% in the nasal lavage; −: subjects with no eosinophils at baseline

^c^Airway Responsiveness FEV_1_ Group +: subjects with a baseline decrease in FEV_1_ of 15% or more during the MBC test; −: subjects with a baseline FEV_1_ decrease < 15%

^d^Airway Responsiveness Resistance Group +: subjects with a baseline increase in resistance of 50% or more between 4 and 16 Hz; −: subjects with a baseline increase in resistance < 50%

^e^not tested because associations exist for structural reasons

*p*: *p*-value of the test comparing the presence or level of a marker in the +risk group and-risk group

During the follow-up, 12 cases of possible asthma were identified (data not shown), based on the presence of asthma-like symptoms and BHR. For 6 of these possible asthma cases, subsequent visits did not confirm the suspicion of asthma. For one subject, possible asthma was detected at both visits 2 and 3 (no visit 4), and for 5 subjects only at visit 4. It concerned 7 hairdressers, 3 pastry makers and 2 bakers. Among them, one hairdresser had a positive reaction to alkaline persulfate, suggesting an occupational asthma.

**Fig. 1:**
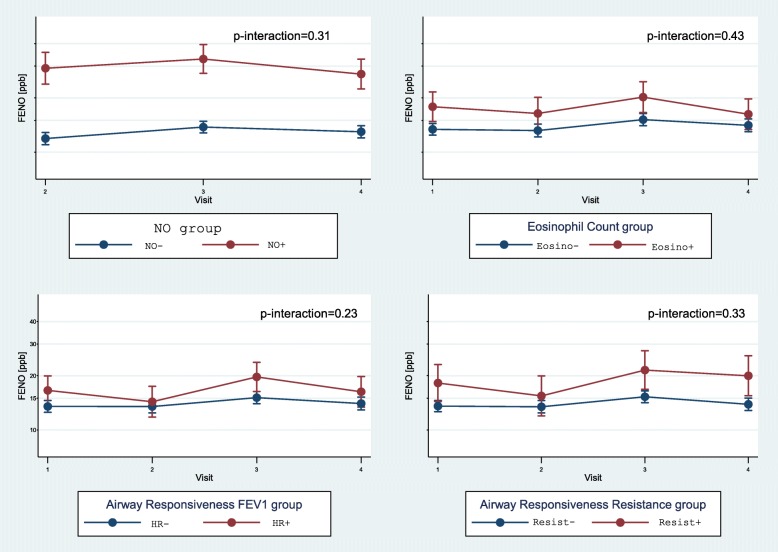
Evolution of FeNO over the visits for each baseline risk group. p-interaction: *p*-value for interaction between the baseline risk group and the number of the visit on the evolution of the FeNO values. NO: Fractional exhaled Nitric Oxide group; Eosino: Eosinophil Count group; HR: Airway Responsiveness FEV_1_ group; Resist: Airway Responsiveness Resistance group

No statistically significant interaction between the risk groups and the visits was observed in any of the modelled markers (see *p*-value for interaction in Table 3). There is therefore no evidence to suggest that the baseline risk groups predict a differential evolution of the airway inflammation and bronchial responsiveness markers, and symptoms considered. Table 3 presents the results obtained from modelling the markers over the four visits according to the baseline risk groups excluding the interaction with the visit. No baseline risk group showed a statistically significant association with the prevalence of symptoms. The two eosinophilic baseline risk groups (NO and Eosino groups) significantly predict subsequent higher proportions of subjects with eosinophils and higher FeNO levels. No other differences in subsequent markers were observed. The HR baseline risk group predicts subsequent steeper decrease of FEV_1_ during MBC test, a higher occurrence of possible asthma cases and higher FeNO levels. The Resist baseline risk group predicts only higher FeNO levels. FeNO levels were higher in all at-risk groups over the course of the follow-up (Fig. 1).

**Table 3:** Associations between risk groups and the evolution of airway inflammation and responsiveness markers among the 318 subjects

Evolution	At inclusion							
	NO Group		Eosinophil Count Group		Airway Responsiveness FEV_1_ Group		Airway Responsiveness Resistance Group	
Number of subjects	+^a^ *n* = 46	-^a^ *n* = 272	+^b^ *n* = 44	-^b^ *n* = 274	+^c^ n = 44	-^c^ n = 274	+^d^ n = 27	-^d^ *n* = 291
Proportion of subjects with rhinoconjunctivitis-like symptoms^e^								
*p*-value for interaction	*p* = 0.26		*p* = 0.13		*p* = 0.57		*p* = 0.33	
Model- predicted value	29.1%	29.0%	32.5%	28.4%	25.2%	29.8%	23.2%	29.7%
OR(p)	1.01 (0.98) ^f^		1.4 (0.48) ^f^		0.70 (0.39) ^f^		0.59 (0.34) ^f^	
Proportion of subjects with asthma-like symptoms^e^								
p-value for interaction	*p* = 0.50		*p* = 0.51		*p* = 0.41		*p* = 0.32	
Model- predicted value	6.5%	8.6%	6.3%	8.7%	8.8%	8.4%	6.3%	8.7%
OR(p)	0.65 (0.56) ^f^		0.62 (0.50) ^f^		1.08 (0.90) ^f^		0.62 (0.55) ^f^	
Percentage decrease during MBC test - FEV_1_[%]^g^								
p-value for interaction	*p* = 0.25		*p* = 0.11		*p* = 0.07		*p* = 0.38	
Model- predicted value	9.7%	9.3%	8.9%	9.4%	**14.1%**	**8.9%**	10.8%	9.2%
Difference (p)	0.44 (0.67) ^h^		0.47 (0.64) ^h^		**5.43 (0.001)** ^**f**^		1.63 (0.19) ^h^	
Percentage increase during MBC test - resistance[%]^g^								
p-value for interaction	*p* = 0.79		*p* = 0.84		*p* = 0.06		*p* = 0.12	
Model- predicted value	22.1%	18.9%	18.9%	19.4%	23.0%	18.7%	25.2%	18.5%
Difference (p)	3.28 (0.17) ^h^		−0.58 (0.80) ^h^		4.24 (0.06) ^h^		6.8 (0.052) ^f^	
Proportion of subjects with a possible asthma^e^								
p-value for interaction	Not estimable		*p* = 0.61		*p* = 0.59		*p* = 0.95	
Model- predicted value	0.9%	1.7%	2.0%	1.6%	**5.0%**	**1.0%**	3.3%	1.5%
OR(p)	0.50 (0.57) ^f^		1.30 (0.79) ^f^		**6.19 (0.012)** ^f^		2.53 (0.36) ^f^	
Proportion of subjects with eosinophil count ≥1%^e^								
p-value for interaction	*p* = 0.94		p = 0.61		*p* = 0.43		p = 0.50	
Model- predicted value	**19.2%**	**10.1%**	**19.6%**	**9.3%**	13.4%	11.5%	10.5%	12.0%
OR(p)	**2.31 (0.003)** ^h^		**2.51 (0.001)** ^f^		1.22 (0.497) ^h^		0.84 (0.66) ^h^	
FeNO level [ppb] ^g^								
p-value for interaction	*p* = 0.31		p = 0.43		*p* = 0.23		p = 0.33	
Model- predicted value	**29.7**	**12.9**	**17.7**	**13.9**	**16.7**	**14.0**	**18.7**	**14.0**
Ratio (p)	**2.30 (< 0.001)** ^f^		**1.27 (0.006)** ^h^		**1.19 (0.038)** ^**h**^		**1.33 (0.007)** ^h^	

^a^NO Group +: subjects with a baseline FeNO level > 27 ppb; −: subjects with a baseline FeNO level < 27 ppb

^b^Eosinophil Count Group +: subjects with a baseline percentage of eosinophils > 1% in the nasal lavage; −: subjects without eosinophils at baseline

^c^Airway Responsiveness FEV_1_ Group +: subjects with a baseline FEV_1_ decrease of 15% or more during the MBC test; −: subjects with a baseline FEV_1_ decrease < 15%

^d^Airway Responsiveness Resistance Group +: subjects with a baseline increase in resistance of 50% or more between 4 and 16 Hz; −: subjects with a baseline resistance increase < 50%

^e^Logistic regression; symptom models adjusted for sex, degree of sensitization, tobacco usage status, training track, and visit; eosinophil count model adjusted for degree of sensitization, training track, and visit

^f^In visits 2,3,4 excluding the interaction between the risk group at inclusion and the number of the visit on the evolution of the marker

^g^Linear regression; adjustment for sex, height, degree of sensitization, tobacco usage status, training track, and visit

^h^In visits 1, 2, 3, 4 excluding interaction between the risk group at inclusion and the number of the visit on the evolution of the marker

*p*-value for interaction: *p*-value for interaction between the baseline risk group and the number of the visit on the evolution of the marker

The results with respect to baseline associations between risk groups and markers were qualitatively similar in the different training tracks, although some differences were no longer statistically significant (see Additional file 2). None of the markers considered, differed in a mixed effect model comparing the three training tracks adjusted on the confounders (data not shown). Evolution of most of the markers is similar according to training track (see Additional file 3). The most striking difference between training tracks is the absence of difference in FeNO levels according to the Eosino group among the hairdressers.

## Discussion

At baseline, the inflammation markers were related to the MBC markers. There was no evidence to suggest that the baseline risk groups predict a differential evolution of the airway inflammation and bronchial responsiveness markers, or the asthma-like symptoms considered. The observed differences between the risk groups in terms of the baseline marker levels persisted during the apprenticeship for most markers; risk groups defined on basis of eosinophil count and FeNO were associated with higher FeNO levels and eosinophil counts during the apprenticeship, but were not associated with higher levels of airway responsiveness markers (FEV_1_ or resistance). On the other hand, the FEV_1_ airway responsiveness risk group predicted higher levels of four markers: FEV_1_ and resistance airway responsiveness, FeNO, and the presence of eosinophils in nasal lavage during the apprenticeship. Finally, FeNO levels were higher in all at-risk groups over the course of the follow-up.

These results were similar in the three training tracks, with the exception of the FeNO levels which were not different according to the Eosino risk group. Twelve possible new asthma cases were identified, only the HR risk group predicted their occurrence.

Thus, only the HR baseline risk group possibly identified vulnerable subjects during the apprenticeship. A single measurement of the other biomarkers used seems to be of limited interest for predicting early stages of asthma.

At baseline, FeNO was associated with a higher FEV_1_ airway responsiveness. This is consistent with the cross-sectional association between FeNO and airway responsiveness that we previously observed in a population of indoor lifeguards [24]. In the present analysis, we also investigated whether the initial FeNO level could predict the development of airway responsiveness during the apprenticeship among apprentices exposed to asthmogens. Our analyses did not confirm this hypothesis. However, longitudinal analyses using this cohort data (13) have shown that an increase in FeNO was associated with the occurrence or aggravation of FEV_1_ airway responsiveness. Thus, those apprentices with a high level of FeNO at the start of the apprenticeship had no greater risk of experiencing BHR during the apprenticeship, but apprentices whose FeNO levels increased between visits were found to be at greater risk of BHR.

In summary, single high levels of FeNO are not in themselves predictive of future airway responsiveness, but the serial measurements of FeNO levels over time may help to focus prevention on subjects at higher risk of developing airway responsiveness, and potentially asthma.

Conversely, having an airway hyperresponsiveness at baseline predicted a consistently high level of FeNO during the apprenticeship. This baseline group predicted the levels of the four makers considered during the apprenticeship, although the strength of the association was low and at the limit of statistical significance for both the increase in resistance during the MBC test and the proportion of subjects with eosinophils. The three baseline groups defined on the basis of an eosinophilic profile (FeNO and/or eosinophils in nasal lavage), predicted only two markers (eosinophils and FeNO) but the strength of this association was higher and had more statistical significance. This eosinophilic profile appeared stable during the apprenticeship.

The levels of eosinophilic markers did not differ between baker and pastry-maker apprentices, who, unlike hairdresser apprentices, are both exposed to HMW agents. This is in line with two clinical studies [9, 25], in which an increase of sputum eosinophils after specific inhalation challenge (SIC) was observed whatever the molecular weight of the agent. Other clinical studies using SIC observed increased amounts of sputum eosinophils in the case of HMW agents [7, 8], or, on the contrary, more eosinophils in the case of LMW agents [6]. An increase in sputum eosinophils is consistent with the fact that most HMW agents cause OA through an IgE-dependent mechanism, whereas many LMW agents induce asthma through a non-IgE-dependent mechanism [26]. The mechanism of asthmogenesis is still unclear for some LMW agents, such as persulfate agents, against which specific IgE antibodies are sometimes found and for which another immunologic mechanism has been suggested [27, 28]. This is consistent with our finding that the FeNO levels did not differ according to the Eosino group among the hairdressers.

The longitudinal relationship between FeNO and airway responsiveness also did not differ according to the training track in this cohort [13]. This is consistent with observations of high levels of FeNO in workers exposed to either sensitizers or irritants [29]. This suggests that mechanisms other than eosinophilic inflammation, such as oxidative stress, might induce an increase in FeNO [24].

No association was found between baseline risk groups and respiratory symptoms, neither at baseline nor during the apprenticeship, although the occurrence of symptoms soon after the start of an apprenticeship has previously been described [10, 30]. This absence of an association might be due to a follow up that was shorter (18 months) than the latency, generally assumed to be 2 to 3 years [10, 30]. However, we identified a small group of 12 subjects with a possible asthma, based on BHR and symptoms. This was reversible for 6 of them. Yet, the HR baseline group predicted the occurrence of these possible asthma cases. Note that our HR group was defined as a decrease of 15% in FEV_1_ rather than the standard 20%. When changing this cutpoint to 20%, the results were similar, but the levels of FeNO were no longer statistically different among the HR group. We think that if our objective is to screen potential future asthma cases, using a less stringent criteria based on 15% decline of FEV_1_ in the MBC test will be more sensitive.

Our study was designed to investigate the early development of airway inflammation and asthma-like symptoms, and thus students with any suspicion of asthma were not included. This might have reduced the statistical power of the study as possible work-exacerbated asthmatics were not included. The participation rate was 24% and the observed drop-out rate was 20% [12]. No difference in the initial clinical and demographic characteristics was observed between subjects lost for follow-up and those who remained in the study, except that the drop-outs were more often smokers. Eighty-five percent of the drop-outs (77/90) were interviewed by telephone regarding their reasons for withdrawing from the study: one third invoked a lack of time and another third invoked departure from the training program (only one student reported allergic conditions). A healthy worker effect seems more likely to play a role when deciding to enrol in an apprenticeship program than when deciding to drop out of it [12, 31]. In the present paper, we considered the evolution in the markers among apprentices follow-up at least twice. It cannot be excluded that the evolution of the markers of the subjects who did not make a second follow up appointment was different from our study subjects. The limited information we got for the drop outs does however not suggest that the evolution is to the worse in these subjects.

## Conclusion

Among this healthy young population, initial BHR predicted consistently high levels of all markers considered and possible asthma. No initial risk group was related to the occurrence of symptoms. A single measurement of the other biomarkers used seems to be of limited interest for predicting early stages of asthma.

## Additional files

Additional file 1: Baseline association between training tracks and the personal characteristics of the 318 subjects. (DOCX 16 kb)

Additional file 2: Baseline associations between risk groups and airway inflammation and responsiveness markers by training track. (DOCX 37 kb)

Additional file 3: Associations between risk groups and the evolution of airway inflammation and responsiveness markers by training track. (DOCX 39 kb)

## Abbreviations

ATS: American thoracic society
BHR: Bronchial hyperresponsiveness
Eosino: Baseline risk group defined by an eosinophil percentage greater than 1% in the nasal lavage
EP: Eosinophil percentage
ERS: European respiratory society
FeNO: Fractional exhaled nitric oxide
FEV_1_: Forced expiratory volum at the first second
FOT: Forced oscillation technique
FVC: Forced vital capacity
HMW: High molecular weight
HR: Baseline risk group defined by a 15% or more decrease in FEV_1_ during the MBC test
IgE: Immunoglobulins E
LMW: Low molecular weight
MBC: Methacholine bronchial challenge
NO: Baseline risk group based on a high level of FeNO
OA: Occupational asthma
Resist: baseline risk group defined by a 50% increase in resistance between 4 and 16 Hz during the MBC test
SIC: Specific inhalation challenge
SPT: Skin prick tests
