# Supplementary material for: Do airway inflammation and airway responsiveness markers at the start of apprenticeship predict their evolution during initial training? A longitudinal study among apprentice bakers, pastry makers and hairdressers
Source: BMC Pulm Med. 2018 Jul 11;18:113. doi: 10.1186/s12890-018-0674-9 (PMC6042364; doi:10.1186/s12890-018-0674-9)
Supplement: Supplementary file 1 — Baseline association between training tracks and the personal characteristics of the 318 subjects. (DOCX 16 kb) [file 12890_2018_674_MOESM1_ESM.docx]

Table S1. Baseline association between training tracks and the personal characteristics of the 318 subjects (median [interquartile range], % (n))

|  | Bakers | Pastry makers | Hairdressers |
| --- | --- | --- | --- |
| Sex: male 55.3% (176) | 103 (93.6%) | 63 (77.8%) | 10 (7.8%) |
| Atopic disposition 37.1% (118) | 31 (28.2%) | 33 (40.7%) | 54 (42.5%) |
| Atopic disease 18.2% (58) | 14 (12.7%) | 17 (21.0%) | 27 (21.3%) |
| Personal atopy based on SPT |  |  |  |
| ≥1 positive response 32.1% (102) | 38 (34.5%) | 32 (39.5%) | 32 (25.2%) |
| Degree of sensitization |  |  |  |
| 0 positive response | 68 (61.8%) | 45 (56.2%) | 87 (69.1%) |
| 1 to 2 positive responses | 22 (20.0%) | 17 (21.2%) | 24 (19.0%) |
| ≥ 3 positive responses | 20 (18.2%) | 18 (22.5%) | 15 (11.9%) |
| Tobacco usage status |  |  |  |
| Non smoker 49.7% (158) | 55 (50.0%) | 41 (50.6%) | 62 (48.8%) |
| Current smoker 46.2% (147) | 51 (46.4%) | 39 (48.1%) | 57 (44.9%) |
| Past smoker 4.1% (13) | 4 (3.6%) | 1 (1.3%) | 8 (6.3%) |
| Baseline FEV_1_ |  |  |  |
| >80.0% 87.1% (277) | 96 (87.3%) | 68 (84.0%) | 113 (89.0%) |
| >70.0% and <80.0% 12.3% (39) | 14 (12.7%) | 11 (13.5%) | 14 (11.0%) |
| <70.0% 0.6% (2) | 0 | 2 (2.5%) | 0 |
| MBC test |  |  |  |
| FEV1 decrease of 15% or more | 12 (10.9%) | 12 (14.8%) | 20 (15.7%) |
| FEV1 decrease of 20% or more | 4 (3.6%) | 5 (6.2%) | 9 (7.1%) |
