# Supplementary material for: Do airway inflammation and airway responsiveness markers at the start of apprenticeship predict their evolution during initial training? A longitudinal study among apprentice bakers, pastry makers and hairdressers
Source: BMC Pulm Med. 2018 Jul 11;18:113. doi: 10.1186/s12890-018-0674-9 (PMC6042364; doi:10.1186/s12890-018-0674-9)
Supplement: Supplementary file 2 — Baseline associations between risk groups and airway inflammation and responsiveness markers by training track. (DOCX 37 kb) [file 12890_2018_674_MOESM2_ESM.docx]

Table S2. Baseline associations between risk groups and airway inflammation and responsiveness markers among the 110 bakers (median [interquartile range], % (n))

|  | NO Group | | Eosinophil Count Group | | | Airway Responsiveness  FEV_1_ Group | | Airway Responsiveness Resistance Group | |
| --- | --- | --- | --- | --- | --- | --- | --- | --- | --- |
|  | +^a^  n=19 | -^a^  n=91 | +^b^  n=15 | -^b^  n=95 | | +^c^  n=12 | -^c^  n=98 | +^d^  n=11 | -^d^  n=99 |
| Rhinoconjunctivitis-like symptoms | 0 | 6.7% (6) | 6.7% (1) | 5.3% (5) | | 0 | 6.1% (6) | 18.2% (2) | 4.0% (4) |
|  | p=0.587 | | p=1.00 | | | p=1.00 | | p=0.110 | |
| Asthma-like symptoms | 10.5% (2) | 2.2% (2) | 13.3% (2) | 2.1% (2) | | 0 | 4.1% (4) | 0 | 4.1% (4) |
|  | p=0.137 | | p=0.089 | | | p=1.00 | | p=1.00 | |
| MBC test |  |  |  |  | |  |  |  |  |
| (pre – post-test) /pretest FEV1[%] | **-9.1**  **[-11.8;-4.6]** | **-6.3**  **[-10.8;-3.6]** | -7.3  [-9.5;-3.7] | -6.7  [-11.0;-4.0] | | *-17.8*  *[-28.3;-16.7]* | *-5.8*  *[-9.2;-3.6]* | **-10.0**  **[-16.7;-7.1]** | **-6.3**  **[-10.7;-3.8]** |
|  | **p=0.0358** | | p=0.724 | | | *^e^* | | **p=0.0342** | |
| (Post– pretest) /pretest Rrs4-16 [%] | 16.7  [12.2;29.7] | 19.0  [9.7;32.8] | 16.7  [7.2;29.7] | 18.8  [11.5;32.8] | | **32.4**  **[17.3;53.8]** | **18.4**  **[9.7;29.7]** | *63.6*  *[52.1;72.3]* | *17.7*  *[9.2;27.2]* |
|  | p=0.604 | | p=0.483 | | | **p=0.025** | | *^e^* | |
| Eosinophil count |  |  |  |  | |  |  |  |  |
| Eosinophil count <1% 86.2% (274) | **57.8% (11)** | **92.3% (84)** |  | *100.0% (95)* | | 91.7% (11) | 85.7% (84) | 90.9% (10) | 85.9% (85) |
|  | **p=0.001** | | *^e^* | | | p=1.00 | | p=1.00 | |
| Eosinophil count [%] if ≥1% | **26.5**  **[14.8;58.3]** | **6.0**  **[3.6;12.0]** | *14.0*  *[5.9;36.4]* |  | | 50.0 (n=1) | 13.0  [5.9;16.7] | 16.7 (n=1) | 13.0  [5.9;36.4] |
|  | **p=0.008** | | *^e^* | | | p=0.247 | | p=0.487 | |
| FeNO [ppb] | *43.9*  *[60.6;33.2]* | *11.1*  *[8.7;15.1]* | **27.6**  **[13.8;56.5]** | | **12.3**  **[8.9;17.2]** | 15.8  [10.6;33.4] | 12.4  [8.9;20.0] | 12.6  [9.5;23.5] | 12.7  [9.0;20.0] |
|  | *^e^* | | **p=0.002** | | | p=0.186 | | p=0.580 | |

^a^: NO Group +: subjects with a baseline FeNO level > 27 ppb; -: subjects with a baseline FeNO level < 27 ppb

^b^: Eosinophil Count Group +: subjects with a baseline percentage of eosinophils > 1% in the nasal lavage; -: subjects with no eosinophils at baseline

^c^: Airway Responsiveness FEV1 Group +: subjects with a baseline decrease in FEV1 of 15% or more during the MBC test; -: subjects with a baseline FEV1 decrease <15%

^d^: Airway Responsiveness Resistance Group +: subjects with a baseline increase in resistance of 50% or more between 4 and 16 Hz; -: subjects with a baseline increase in resistance < 50%

^e^: not tested because associations exist for structural reasons

p: p-value of the test comparing the presence or level of a marker in the +risk group and-risk group

Table S2. Baseline associations between risk groups and airway inflammation and responsiveness markers among the 81 pastry makers (median [interquartile range], % (n))

|  | NO Group | | Eosinophil Count Group | | | Airway Responsiveness  FEV_1_ Group | | Airway Responsiveness Resistance Group | |
| --- | --- | --- | --- | --- | --- | --- | --- | --- | --- |
|  | +^a^  n=13 | -^a^  n=68 | +^b^  n=14 | -^b^  n=67 | | +^c^  n=12 | -^c^  n=69 | +^d^  n=9 | -^d^  n=72 |
| Rhinoconjunctivitis-like symptoms | 15.4% (2) | 8.8% (6) | 14.3% (2) | 9.0% (6) | | 8.3% (1) | 10.1% (7) | 11.1% (1) | 9.7% (7) |
|  | p=0.378 | | p=0.419 | | | p=0.662 | | p=0.628 | |
| Asthma-like symptoms | 7.7% (1) | 1.5% (1) | 0.0% (0) | 3.0% (2) | | 0 | 2.9% (2) | 11.1% (1) | 1.4% (1) |
|  | p=0.297 | | p=0.682 | | | p=0.724 | | p=0.211 | |
| MBC test |  |  |  |  | |  |  |  |  |
| (pre – post-test) /pretest FEV1[%] | -10.3  [-15.0;-5.4] | -5.7  [-9.9;-2.7] | -5.2  [-8.4;-3.0] | -5.8  [-11.2;-3.1] | | *-18.9*  *[-24.4;-16.8]* | *-5.5*  *[-8.0;-2.6]* | -9.8  [-13.1;-5.7] | -5.7  [-10.1;-3.0] |
|  | p=0.097 | | p=0.446 | | | **^e^** | | p=0.286 | |
| (Post– pretest) /pretest Rrs4-16 [%] | 25.0  [12.2;42.6] | 13.3  [4.9;30.8] | 12.9  [6.2;29.4] | 15.0  [4.6;34.9] | | 12.5  [7.3;27.5] | 15.0  [4.6;34.6] | *67.4*  *[54.1;78.1]* | *11.9*  *[4.5;25.2]* |
|  | p=0.177 | | p=0.764 | | | p=0.710 | | *^e^* | |
| Eosinophil count |  |  |  |  | |  |  |  |  |
| Eosinophil count <1% 86.2% (274) | 69.2% (9) | 85.3% (58) |  | *100.0% (67)* | | 75.0% (9) | 84.1% (58) | 100.0% (9) | 80.6% (58) |
|  | p=0.157 | | *^e^* | | | p=0.342 | | p=0.164 | |
| Eosinophil count [%] if ≥1% | 42.5  [24.8;64.0] | 13.9  [7.0;24.0] | *20.5*  *[9.8;33.3]* |  | | 24.0  [21.0;71.4] | 14.0  [7.0;33.3] | 20.5  [9.8;33.3] | - |
|  | p=0.0660 | | *^e^* | | | p=0.243 | |  | |
| FeNO [ppb] | *56.4*  *[37.6;64.1]* | *13.5*  *[9.5;17.4]* | **21.6**  **[13.9;50.8]** | | **14.4**  **[9.6;20.4]** | **22.6**  **[18.7;34.7]** | **14.0**  **[9.6;18.8]** | 19.9  [15.4;34.8] | 14.8  [10.0;21.3] |
|  | *^e^* | | **p=0.0405** | | | **p=0.0055** | | p=0.383 | |

^a^: NO Group +: subjects with a baseline FeNO level > 27 ppb; -: subjects with a baseline FeNO level < 27 ppb

^b^: Eosinophil Count Group +: subjects with a baseline percentage of eosinophils > 1% in the nasal lavage; -: subjects with no eosinophils at baseline

^c^: Airway Responsiveness FEV1 Group +: subjects with a baseline decrease in FEV1 of 15% or more during the MBC test; -: subjects with a baseline FEV1 decrease <15%

^d^: Airway Responsiveness Resistance Group +: subjects with a baseline increase in resistance of 50% or more between 4 and 16 Hz; -: subjects with a baseline increase in resistance < 50%

^e^: not tested because associations exist for structural reasons

p: p-value of the test comparing the presence or level of a marker in the +risk group and-risk group

Table S2. Baseline associations between risk groups and airway inflammation and responsiveness markers among the 127 hairdressers (median [interquartile range], % (n))

|  | NO Group | | Eosinophil Count Group | | | Airway Responsiveness  FEV_1_ Group | | Airway Responsiveness Resistance Group | |
| --- | --- | --- | --- | --- | --- | --- | --- | --- | --- |
|  | +^a^  n=14 | -^a^  n=113 | +^b^  n=15 | -^b^  n=112 | | +^c^  n=20 | -^c^  n=107 | +^d^  n=7 | -^d^  n=120 |
| Rhinoconjunctivitis-like symptoms | 7.1% (1) | 11.5% (13) | 6.7% (1) | 11.6% (13) | | 5.0% (1) | 12.1% (13) | 0.0% (0) | 11.7% (14) |
|  | p=0.524 | | p=0.484 | | | p=0.312 | | p=0.432 | |
| Asthma-like symptoms | 0.0% (0) | 1.8% (2) | 0.0% (0) | 1.8% (2) | | 0 | 1.9% (2) | 0.0% (0) | 1.7% (2) |
|  | p=0.797 | | p=0.777 | | | p=0.709 | | p=0.892 | |
| MBC test |  |  |  |  | |  |  |  |  |
| (pre – post-test) /pretest FEV1[%] | **-12.8**  **[-17.9;-5.4]** | **-5.9**  **[-10.1;-3.2]** | -9.9  [-19.1;-5.9] | -5.8  [-11.3;-3.3] | | *-19.3*  *[-26.0;-18.1]* | *-5.4*  *[-8.7;-2.6]* | **-9.6**  **[-19.1;-8.4]** | **-5.9**  **[-11.8;-3.1]** |
|  | **p=0.040** | | p=0.063 | | | **^e^** | | **p=0.018** | |
| (Post– pretest) /pretest Rrs4-16 [%] | 17.4  [5.1;40.9] | 13.7  [5.6;21.7] | 13.8  [4.5;23.5] | 13.8  [6.2;22.3] | | **26.8**  **[15.8;36.9]** | **11.6**  **[5.0;21.2]** | *56.4*  *[51.3;90.8]* | *12.9*  *[5.1;21.2]* |
|  | p=0.336 | | p=0.852 | | | **p=0.0010** | | *^e^* | |
| Eosinophil count |  |  |  |  | |  |  |  |  |
| Eosinophil count <1% 86.2% (274) | 85.7% (12) | 88.5% (100) |  | *100.0% (112)* | | 80.0% (16) | 89.7% (96) | 71.4% (5) | 89.2% (107) |
|  | p=0.516 | | *^e^* | | | p=0.190 | | p=0.193 | |
| Eosinophil count [%] if ≥1% | 31.1  [2.2;60.0] | 6.2  [2.9;20.0] | *6.2*  *[2.2;26.6]* |  | | 34.5  [5.7;70.0] | 470  [2.0;20.0] | 70.0  [60.0;80.0] | 4.7  [2.2;10.2] |
|  | p=0.865 | | *^e^* | | | p=0.296 | | p=0.062 | |
| FeNO [ppb] | *40.4*  *[32.1;70.2]* | *11.3*  *[7.7;14.5]* | 11.1  [7.3;17.2] | | 12.1  [8.6;17.2] | 14.9  [8.8;24.0] | 11.8  [8.2;16.5] | **19.4**  **[11.1;70.9]** | **11.9**  **[8.1;16.8]** |
|  | *^e^* | | p=0.849 | | | p=0.233 | | **p=0.027** | |

^a^: NO Group +: subjects with a baseline FeNO level > 27 ppb; -: subjects with a baseline FeNO level < 27 ppb

^b^: Eosinophil Count Group +: subjects with a baseline percentage of eosinophils > 1% in the nasal lavage; -: subjects with no eosinophils at baseline

^c^: Airway Responsiveness FEV1 Group +: subjects with a baseline decrease in FEV1 of 15% or more during the MBC test; -: subjects with a baseline FEV1 decrease <15%

^d^: Airway Responsiveness Resistance Group +: subjects with a baseline increase in resistance of 50% or more between 4 and 16 Hz; -: subjects with a baseline increase in resistance < 50%

^e^: not tested because associations exist for structural reasons

p: p-value of the test comparing the presence or level of a marker in the +risk group and-risk group
