# Supplementary material for: Do airway inflammation and airway responsiveness markers at the start of apprenticeship predict their evolution during initial training? A longitudinal study among apprentice bakers, pastry makers and hairdressers
Source: BMC Pulm Med. 2018 Jul 11;18:113. doi: 10.1186/s12890-018-0674-9 (PMC6042364; doi:10.1186/s12890-018-0674-9)
Supplement: Supplementary file 3 — Associations between risk groups and the evolution of airway inflammation and responsiveness markers by training track. (DOCX 39 kb) [file 12890_2018_674_MOESM3_ESM.docx]

Table S3. Associations between risk groups and the evolution of airway inflammation and responsiveness markers among the 110 bakers.

| At inclusion  Evolution | NO Group | | Eosinophil Count Group | | Airway Responsive-  ness FEV_1_ Group | | Airway Responsiveness Resistance Group | |
| --- | --- | --- | --- | --- | --- | --- | --- | --- |
| Number of subjects | +^a^  n=19 | -^a^  n=191 | +^b^  n=15 | -^b^  n=95 | +^c^  n=12 | -^c^  n=98 | +^d^  n=11 | -^d^  n=99 |
| Proportion of subjects with rhinoconjunctivitis-like symptoms^e^ |  |  |  |  |  |  |  |  |
| p-value for interaction | p=0.16 | | p=0.12 | | p=0.77 | | p=0.24 | |
| Model- predicted value | 34.1% | 28.0% | 28.6% | 29.2% | 13.6% | 31.2% | 29.2% | 29.1% |
| OR(p) | 1.62 (0.52) ^f^ | | 0.95 (0.95) ^f^ | | 0.19 (0.09) ^f^ | | 1.00 (0.99) ^f^ | |
| Proportion of subjects with asthma-like symptoms^e^ |  | |  | |  | |  | |
| p-value for interaction | not estimable | | p=0.25 | | not estimable | | not estimable | |
| Model- predicted value |  |  | 13.9% | 8.9% | 6.0% | 9.8% |  |  |
| OR(p) |  |  | 2.58 (0.56) ^f^ | | 0.39 (0.64) ^f^ | |  |  |
| Percentage decrease during MBC test - FEV1[%]^g^ |  |  |  |  |  |  |  |  |
| p-value for interaction | p=0.26 | | p=0.62 | | p=0.55 | | p= 0.17 | |
| Model- predicted value | 9.5% | 9.7% | 9.1% | 9.7% | **14.1%** | **9.8%** | 11.6% | 9.4% |
| Difference (p) | 0.38 (0.81) ^h^ | | 0.59 (0.71) ^h^ | | **4.36** **(0.03)^f^** | | 2.20 (0.23) ^h^ | |
| Percentage increase during MBC test - resistance[%]^g^ |  |  |  |  |  |  |  |  |
| p-value for interaction | p=0.25 | | p=0.58 | | p=0.22 | | p= 0.15 | |
| Model- predicted value | 18.3% | 23.4% | 19.5% | 23.1% | **30.4%** | **21.5%** | **36.9%** | **20.7%** |
| Difference (p) | 5.1 (0.19) ^h^ | | 3.58 (0.39) ^h^ | | **8.9 (0.04) ^h^** | | **16.1 (0.003) ^f^** | |
| Proportion of subjects with eosinophil count ≥1% ^e^ |  |  |  |  |  |  |  |  |
| p-value for interaction | p=0.34 | | p=0.43 | | p=0.42 | | p=0.84 | |
| Model- predicted value | **23.2%** | **9.6%** | 21.6% | 10.1% | not estimable | | not estimable | |
| OR(p) | **2.99 (0.005)** ^h^ | | 2.52 (0.06) ^f^ | | 0.59 (0.38) ^h^ | | 0.71 (0.59) ^h^ | |
| FeNO level [ppb] ^g^ |  |  |  |  |  |  |  |  |
| p-value for interaction | p=0.14 | | p=0.84 | | p=0.14 | | p=0.35 | |
| Model- predicted value | **28.2** | **14.3** | **23.3** | **14.5** | 16.7 | 15.3 | 20.1 | 15.0 |
| Ratio (p) | **1.97 (<0.001)** ^f^ | | **1.61 (0.001) ^h^** | | 1.10 (0.60) ^h^ | | 1.34 (0.10) ^h^ | |

^a^: NO Group +: subjects with a baseline FeNO level > 27 ppb; -: subjects with a baseline FeNO level < 27 ppb

^b^: Eosinophil Count Group +: subjects with a baseline percentage of eosinophils > 1% in the nasal lavage; -: subjects without eosinophils at baseline

^c^: Airway Responsiveness FEV1 Group +: subjects with a baseline FEV1 decrease of 15% or more during the MBC test; -: subjects with a baseline FEV1 decrease <15%

^d^: Airway Responsiveness Resistance Group +: subjects with a baseline increase in resistance of 50% or more between 4 and 16 Hz; -: subjects with a baseline resistance increase < 50%

^e^: logistic regression; symptom models adjusted for sex, degree of sensitization, tobacco usage status, training track, and visit; eosinophil count model adjusted for degree of sensitization, training track, and visit

^f^: in visits 2,3,4 excluding the interaction between the risk group at inclusion and the number of the visit on the evolution of the marker

^g^: linear regression; adjustment for sex, height, degree of sensitization, tobacco usage status, training track, and visit

^h^: in visits 1, 2, 3, 4 excluding interaction between the risk group at inclusion and the number of the visit on the evolution of the marker

p-value for interaction: p-value for interaction between the baseline risk group and the number of the visit on the evolution of the marker.

Table S3. Associations between risk groups and the evolution of airway inflammation and responsiveness markers among the 81 pastry makers.

| At inclusion  Evolution | NO Group | | Eosinophil Count Group | | Airway Responsive-  ness FEV_1_ Group | | Airway Responsiveness Resistance Group | |
| --- | --- | --- | --- | --- | --- | --- | --- | --- |
| Number of subjects | +^a^  n=13 | -^a^  n=68 | +^b^  n=14 | -^b^  n=67 | +^c^  n=12 | -^c^  n=69 | +^d^  n=9 | -^d^  n=72 |
| Proportion of subjects with rhinoconjunctivitis-like symptoms^e^ |  |  |  |  |  |  |  |  |
| p-value for interaction | p=0.82 | | p=0.61 | | p=0.47 | | p=0.82 | |
| Model- predicted value | 13.8% | 25.9% | 32.6% | 21.1% | 14.2% | 25.8% | 9.2% | 25.4% |
| OR(p) | 0.20 (0.29) ^f^ | | 3.34 (0.25) ^f^ | | 0.23 (0.22) ^f^ | | 0.10 (0.16) ^f^ | |
| Proportion of subjects with asthma-like symptoms^e^ |  | |  | |  | |  | |
| p-value for interaction | p= 0.26 | | not estimable | | not estimable | | p= 0.83 | |
| Model- predicted value | not estimable | |  |  |  |  | not estimable | |
| OR(p) | 2.40 (0.41)^f^ | | 0.56 (0.61)^f^ | | 0.58 (0.63)^f^ | | 4.3 (0.07)^f^ | |
| Percentage decrease during MBC test - FEV1[%]^g^ |  |  |  |  |  |  |  |  |
| p-value for interaction | p=0.98 | | p=0.63 | | **p=0.001** | | p= 0.34 | |
| Model- predicted value | 8.6% | 8.5% | 6.7% | 8.9% | **16.0%** | **7.1%** | 10.8% | 8.2% |
| Difference (p) | 0.13 (0.96) ^h^ | | 2.18 (0.24) ^h^ | | **8.81 (<0.001)^f^** | | 2.57 (0.26) ^h^ | |
| Percentage increase during MBC test - resistance[%]^g^ |  |  |  |  |  |  |  |  |
| p-value for interaction | p=0.73 | | p=0.70 | | p=0.95 | | p= 0.83 | |
| Model- predicted value | 24.6% | 17.2% | 17.4% | 18.5% | 16.3% | 18.8% | 12.4% | 17.8% |
| Difference (p) | 7.40 (0.11) ^h^ | | 1.15 (0.75) ^h^ | | 2.51 (0.49) ^h^ | | 5.40 (0.31) ^f^ | |
| Proportion of subjects with eosinophil count ≥1% ^e^ |  |  |  |  |  |  |  |  |
| p-value for interaction | p=0.99 | | p=0.37 | | p=0.77 | | p=0.78 | |
| Model- predicted value | 21.2% | 15.0% | **26.8%** | **12.9%** | not estimable | | not estimable | |
| OR(p) | 1.64 (0.44) ^h^ | | **2.62 (0.04) ^f^** | | 2.07 (0.15) ^h^ | | 0.25 (0.11) ^h^ | |
| FeNO level [ppb] ^g^ |  |  |  |  |  |  |  |  |
| p-value for interaction | p=0.42 | | p=0.10 | | p=0.11 | | p=0.57 | |
| Model- predicted value | **33.8** | **14.0** | 18.9 | 15.4 | **21.5** | **15.0** | 18.6 | 15.6 |
| Ratio (p) | **2.41 (<0.001)** ^f^ | | 1.22 (0.16) ^h^ | | **1.43 (0.01)^h^** | | 1.19 (0.31) ^h^ | |

^a^: NO Group +: subjects with a baseline FeNO level > 27 ppb; -: subjects with a baseline FeNO level < 27 ppb

^b^: Eosinophil Count Group +: subjects with a baseline percentage of eosinophils > 1% in the nasal lavage; -: subjects without eosinophils at baseline

^c^: Airway Responsiveness FEV1 Group +: subjects with a baseline FEV1 decrease of 15% or more during the MBC test; -: subjects with a baseline FEV1 decrease <15%

^d^: Airway Responsiveness Resistance Group +: subjects with a baseline increase in resistance of 50% or more between 4 and 16 Hz; -: subjects with a baseline resistance increase < 50%

^e^: logistic regression; symptom models adjusted for sex, degree of sensitization, tobacco usage status, training track, and visit; eosinophil count model adjusted for degree of sensitization, training track, and visit

^f^: in visits 2,3,4 excluding the interaction between the risk group at inclusion and the number of the visit on the evolution of the marker

^g^: linear regression; adjustment for sex, height, degree of sensitization, tobacco usage status, training track, and visit

^h^: in visits 1, 2, 3, 4 excluding interaction between the risk group at inclusion and the number of the visit on the evolution of the marker

p-value for interaction: p-value for interaction between the baseline risk group and the number of the visit on the evolution of the marker.

Table S3. Associations between risk groups and the evolution of airway inflammation and responsiveness markers among the 127 hairdressers.

| At inclusion  Evolution | NO Group | | Eosinophil Count Group | | Airway Responsive-  ness FEV_1_ Group | | Airway Responsiveness Resistance Group | |
| --- | --- | --- | --- | --- | --- | --- | --- | --- |
| Number of subjects | +^a^  n=14 | -^a^  n=113 | +^b^  n=15 | -^b^  n=112 | +^c^  n=9 | -^c^  n=118 | +^d^  n=20 | -^d^  n=107 |
| Proportion of subjects with rhinoconjunctivitis-like symptoms^e^ |  |  |  |  |  |  |  |  |
| p-value for interaction | p=0.07 | | p=0.19 | | p=0.71 | | p=0.54 | |
| Model- predicted value | 32.1% | 33.1% | 30.9% | 33.3% | 40.9% | 31.5% | 34.6% | 32.9% |
| OR(p) | 0.94 (0.92) ^f^ | | 0.86 (0.80) ^f^ | | 1.75 (0.26) ^f^ | | 1.11 (0.90) ^f^ | |
| Proportion of subjects with asthma-like symptoms^e^ |  | |  | |  | |  | |
| p-value for interaction | p=0.67 | | p=0.96 | | p=0.43 | | not estimable | |
| Model- predicted value | 8.1% | 13.5% | 5.2% | 13.9% | 16.6% | 12.5% | 10.0% | 13.2% |
| OR(p) | 0.46 (0.47) ^f^ | | 0.23 (0.23) ^f^ | | 1.62 (0.53) ^f^ | | 0.66 (0.77) ^f^ | |
| Percentage decrease during MBC test - FEV1[%]^g^ |  |  |  |  |  |  |  |  |
| p-value for interaction | p=0.08 | | p=0.16 | | p=0.56 | | p= 0.31 | |
| Model- predicted value | 10.3% | 9.5% | 10.7% | 9.4% | **13.3%** | **9.0%** | 11.4% | 9.4% |
| Difference (p) | 0.78 (0.66) ^h^ | | 1.29 (0.46) ^h^ | | **4.32** **(0.004)^f^** | | 2.0 (0.40) ^h^ | |
| Percentage increase during MBC test - resistance[%]^g^ |  |  |  |  |  |  |  |  |
| p-value for interaction | p=0.35 | | p=0.84 | | **p=0.005** | | p= 0.44 | |
| Model- predicted value | **26.8%** | **16.0%** | 22.1% | 16.5% | **22.9%** | **16.0%** | 27.1% | 16.7% |
| Difference (p) | **10.85 (0.008) ^h^** | | 5.57 (0.16) ^h^ | | **6.8 (0.05) ^h^** | | 10.4 (0.15) ^f^ | |
| Proportion of subjects with eosinophil count ≥1% ^e^ |  |  |  |  |  |  |  |  |
| p-value for interaction | p=0.26 | | p=0.15 | | p=0.65 | | p=0.67 | |
| Model- predicted value | **15.9%** | **7.2%** | 12.8% | 6.4% | 9.4% | 8.2% | 17.7% | 7.7% |
| OR(p) | **2.63 (0.04)** ^h^ | | 2.27 (0.13) ^f^ | | 1.18 (0.74) ^h^ | | 2.83 (0.09) ^h^ | |
| FeNO level [ppb] ^g^ |  |  |  |  |  |  |  |  |
| p-value for interaction | p=0.08 | | p=0.99 | | p=0.93 | | p=0.24 | |
| Model- predicted value | **32.2** | **11.2** | 12.7 | 12.6 | 14.0 | 12.4 | **21.7** | **12.3** |
| Ratio (p) | **2.87 (<0.001)** ^f^ | | 1.00 (0.98) ^h^ | | 1.13 (0.36) ^h^ | | **1.77 (0.006)** ^h^ | |

^a^: NO Group +: subjects with a baseline FeNO level > 27 ppb; -: subjects with a baseline FeNO level < 27 ppb

^b^: Eosinophil Count Group +: subjects with a baseline percentage of eosinophils > 1% in the nasal lavage; -: subjects without eosinophils at baseline

^c^: Airway Responsiveness FEV1 Group +: subjects with a baseline FEV1 decrease of 15% or more during the MBC test; -: subjects with a baseline FEV1 decrease <15%

^d^: Airway Responsiveness Resistance Group +: subjects with a baseline increase in resistance of 50% or more between 4 and 16 Hz; -: subjects with a baseline resistance increase < 50%

^e^: logistic regression; symptom models adjusted for sex, degree of sensitization, tobacco usage status, training track, and visit; eosinophil count model adjusted for degree of sensitization, training track, and visit

^f^: in visits 2,3,4 excluding the interaction between the risk group at inclusion and the number of the visit on the evolution of the marker

^g^: linear regression; adjustment for sex, height, degree of sensitization, tobacco usage status, training track, and visit

^h^: in visits 1, 2, 3, 4 excluding interaction between the risk group at inclusion and the number of the visit on the evolution of the marker

p-value for interaction: p-value for interaction between the baseline risk group and the number of the visit on the evolution of the marker.
